# The role of the private sector in delivering essential packages of health services: lessons from country experiences

**DOI:** 10.1136/bmjgh-2022-010742

**Published:** 2023-01-18

**Authors:** Sameen Siddiqi, Wafa Aftab, A Venkat Raman, Agnès Soucat, Ala Alwan

**Affiliations:** 1 Department of Community Health Sciences, Aga Khan University, Karachi, Pakistan; 2 Department of Global Public Health and Primary Care, University of Bergen, Bergen, Norway; 3 Faculty of Management Studies, University of Delhi, New Delhi, India; 4 Division of Health and Social Protection, France Development Agency (AFD), Paris, France; 5 London School of Hygiene & Tropical Medicine, London, UK

**Keywords:** Health policy, Health services research, Health systems

## Abstract

Many countries are adopting essential packages of health services (EPHS) to implement universal health coverage (UHC), which are mostly financed and delivered by the public sector, while the potential role of the private health sector (PHS) remains untapped. Currently, many low-income and lower middle-income countries (LLMICs) have devised EPHS; however, guidance on translating these packages into quality, accessible and affordable services is limited. This paper explores the role of PHS in achieving UHC, identifies key concerns and presents the experience of the Diseases Control Priorities 3 Country Translation project in Afghanistan, Ethiopia, Pakistan, Somalia, Sudan and Zanzibar. There are key challenges to engagement of the PHS, which include the complexity and heterogeneity of private providers, their operation in isolation of the health system, limitations of population coverage and equity when left to PHS’s own choices, and higher overall cost of care for privately delivered services. Irrespective of the strategies employed to involve the PHS in delivering EPHS, it is necessary to identify private providers in terms of their characteristics and contribution, and their response to regulatory tools and incentives. Strategies for regulating private providers include better statutory control to prevent unlicensed practice, self-regulation by professional bodies to maintain standards of practice and accreditation of large private hospitals and chains. Potentially, purchasing delivery of essential services by engaging private providers can be an effective ‘regulatory approach’ to modify provider behaviour. Despite existing experience, more research is needed to better explore and operationalise the role of PHS in implementing EPHS in LLMICs.

**Summary box**

- Private sector is a major provider of health services in many low-income and middle-income countries, yet it frequently operates on objectives that are self-guided and market-oriented and is not aligned with public sector goals including universal health coverage.
- In a health system where the private health sector is providing a major part of essential health services, implementing the essential packages of health services without its involvement seems unrealistic.
- While there is growing guidance on developing universal health coverage packages of health services, the role of private sector in implementing these packages is generally missing. Addressing this gap is critical for the transition from package design to effective implementation.
- Governments need to address key barriers related to governance, regulation, accountability and quality of services, guided by existing evidence, international experience and lessons learnt.

## Introduction

Private health sector (PHS) providers are a major actor for provision of health services in low-income and lower middle-income countries (LLMICs). While they operate primarily with commercial and market-oriented motives, there is an enormous scope for them to play a key role in the progress towards achieving universal health coverage (UHC) in most countries.

According to the WHO, UHC means that ‘all people have access to the health services they need, when and where they need them, without financial hardship’.^1^ Many countries are using essential packages of health services (EPHS) to progressively implement UHC. An EPHS ‘comprises those healthcare services that the government is providing or is aspiring to provide to its citizens in an equitable manner. Equity involves equal coverage across population groups, adequate physical access to services for all and adequate financial protection, particularly for the poor’.^2^ While these packages are mostly designed and partly delivered by the public sector, the potential role of the PHS that delivers a significant proportion of these services is still untapped. Increasingly normative and practical guidance on the development of these packages is available to countries.^3 4^ Processes of deliberation for development of benefit packages are maturing, and the need for institutionalisation of the process at national and subnational levels is being increasingly asserted.^5 6^ According to Glassman and Chalkidou,^7^ at least 64 low-income and middle-income countries have devised explicit EPHS and the number is progressively increasing particularly after the endorsement of UHC as a target in the Sustainable Development Goals.

However, guidance on how to translate an EPHS through effective implementation into quality, accessible and affordable healthcare services is limited. The current literature on country experiences tells us little about how to align the objectives and interests of various actors, especially the PHS, to implement EPHS and accelerate progress towards UHC. The contexts across LLMICs where these packages need to be implemented are diverse and elude attempts at standardisation of implementation approaches. This contrasts with the relatively more standard approaches now available for designing UHC packages and deciding on priority health services.^8 9^

Many LLMICs where EPHS are currently being implemented have complex, mixed health systems. Along with a public sector of varying capacity and breadth, these countries often have an extensive and heterogenous PHS, with varying degrees of governance effectiveness. This mixed structure of the health system means that it may not be possible to provide universal access to essential health services without effective involvement of the private sector; at the same time, engaging this sector in the provision of publicly funded packages raises key questions of accountability, quality, efficiency, organisational capacity and governance,^8 10^ which are yet to be appropriately answered.

We argue that the delivery of services by the PHS must be broadly understood within the context of the overall health system rather than just the private providers in isolation.^11^ A comprehensive plan for achieving universal access to health services should strategically review the role of public and private sectors in service provision so that the two complement each other in achieving health sector goals not only of universal coverage but also of health security learning from the recent experience that the world has confronted as a result of the COVID-19 pandemic.

In this paper, drawing on existing literature and review of country experiences, we explore the role that the PHS could play in achieving UHC, present the experience of the six countries in engaging this sector and identify key areas of concern and how they might be approached systematically while implementing EPHS. We conducted electronic searches in Medline and Google Scholar, performed a forward citation search of studies which cited the included articles and included further articles after consultation among coauthors based on experience. The primary theme was to review the role of the private sector in provision of essential health services or UHC. Our search was limited to publications in English.

## Typology and characteristics of private sector providers in mixed health systems

In many LLMICs, a key barrier to a policy approach to the PHS is the inability of policy-makers and planners to accurately characterise it. This is because the sector is often heterogenous and provides a broad array of services from small shops selling medicines to independent practitioners, including unlicensed providers, to large corporate hospitals and private insurers.^12^ Different types of providers serve different types of populations, provide different kinds of services and most importantly require different regulatory strategies to better align their activities with the overall goals of the health system.^11^ Strategically leveraging the role of the PHS should start with an assessment of the sector’s diversity, composition and contribution.^13–15^ While it is challenging to classify private providers in well-defined categories in LLMICs, in this paper we have adapted the categories of private providers as defined by McPake and Hanson^11^ (Table 1).

**Table 1:** Typology of private health sector providers in low-income and middle-income countries

Category	Description
Unqualified and underqualified providers	These are sometimes the main providers of health services to poor people. They include outlets such as traditional healers, faith healers, non-qualified or unlicensed caregivers, non-formulary-based drugs shops.
Not-for-profit providers	This is a heterogenous group of providers that include large non-governmental organisations, faith-based providers or donor-funded organisations. These have frequently been contracted to provide services such as family planning or primary care in specific locations or to reach out to disadvantaged populations.
Formally registered small-to-medium private practices	In some LLMICs, such practices make up a large proportion of the private health sector. They usually provide fee-for-service clinical interventions; however, their quality and cost-effectiveness may be questionable, and they normally exclude those who cannot pay. Strategic purchasing or social franchising for special package of services may be options for the government to influence the range and quality of services.^11 41 42^
Corporate commercial hospital sector	Although rapidly growing, it still plays a minor part in provision of health services in LLMICs, even where it is well developed. The cost of health services provided makes them inaccessible for most LLMICs households. While these hospitals provide good quality services to the affluent population, their role in achieving universal access to services is limited because large-scale purchasing cannot be undertaken.^11^

LLMICs, low-income and lower middle-income countries.

## The role of private sector in implementation of EPHS

Much of the existing literature on EPHS focuses on package development. Much less information is available on country experiences regarding implementation of these packages and even less on the role of the PHS, except in certain areas such as health insurance and commodity supply.^16^ More pertinent information is available regarding public–private partnerships (PPP) through outsourcing of publicly financed health services to the PHS, although it is often not specific to the delivery of EPHS.^17–19^ Previous experience on implementing EPHS comes mostly from countries that are in crisis and those in postconflict states that receive significant donor funding for health, such as in Afghanistan,^20^ Cambodia,^21^ East Timor, Mozambique and Uganda.^22^ Two illustrative examples from Afghanistan and Cambodia are briefly discussed.

Around the year 2000, Afghanistan had some of the world’s worst health indicators and a devastated health system. The public health sector was largely dysfunctional, with services delivered by a multitude of national and international non-governmental organisations (NGOs). In parallel to the development of their EPHS (called the basic package of health services) in 2003, a decision was made by the Ministry of Public Health (MOPH) to contract NGOs to provide these services.^20–23^ Despite concerns that health service delivery was a function of the state, the donors encouraged contracting with well-established NGOs for provision of EPHS in defined geographic areas.^24^ The NGOs were paid according to budgets they submitted, with full payment depending on achievement of agreed-on goals. The institutionalisation of a grants and contracts management unit within the MOPH allowed the Ministry to lead the nationwide implementation of EPHS, which was instrumental in increasing access especially for women and increased use of services for birth deliveries.^20^

In 1999, in Cambodia, management of public sector primary care facilities was contracted out to NGOs in five randomly selected districts.^21^ The contracts specified targets for maternal and child health service improvement. The programme increased the availability of 24-hour services, reduced provider absence and increased supervisory visits. It involved increased public health funding and led to offsetting reductions in private expenditure as residents in treated districts switched from unlicenced drug sellers and traditional healers to government clinics. Concurrently, the Asian Development Bank piloted two models of contracting for health services: (1) contracting out, where contractors had full responsibility for delivery of all district health services in accordance with the Health Coverage Plan and (2) contracting in, where contractors only managed district healthcare services, with staff remaining MOH civil servants. An evaluation found that contracting to NGOs was feasible, cost-effective, high performing and equitable and effectively targeted and benefited the poor.^25^

## Feasibility of engaging the private sector in implementing EPHS: experience from six countries

More recently, the Diseases Control Priorities 3 (DCP3) Country Translation Project^26^ conducted a review of the experience of Afghanistan, Ethiopia, Pakistan, Somalia, Sudan and Zanzibar^27–32^ in setting and implementing EPHS using the DCP3 evidence and model packages. All countries have a mix of public and private providers. Formally registered providers operating as individuals or small to medium facilities seem to provide the bulk of services in the private sector, especially in urban areas. Despite its importance, the PHS does not play a major role in the delivery of EPHS. As mentioned, Afghanistan is an outlier, where most of the essential package of health services is delivered by NGOs through outsourcing of services. Notwithstanding its short-term benefits, outsourcing is unlikely to be sustainable because of the unpredictability and increasing scarcity of external aid for health.^33^

There is a wide range of private healthcare providers from large tertiary hospitals, qualified practitioners to unqualified providers in all six countries. In all countries, policy and regulatory frameworks exist to varying degrees to govern the PHS, but no country, including those with social health insurance programmes, is using this sector in the delivery of EPHS.

All countries, except Ethiopia, have policy frameworks that support PPP. The predominant mechanism for engagement of the PHS is contracting. PPPs are being used in Afghanistan, Pakistan and Somalia to enhance delivery of services. There is only limited use of social marketing and franchising in delivery of the EPHS, except for services such as family planning in Pakistan and family planning and nutrition in Afghanistan, but they are being actively considered in Zanzibar. Out-of-pocket expenditure as a percent of total health expenditure is substantial in all countries except Zanzibar, where it is less than 20%.

Table 2 summarises the feasibility of engaging the private sector in the implementation of EPHS and presents information on related health financing and service use indicators in the six focus countries.

**Table 2:** Status of the private health sector arrangements in the six countries and feasibility of engagement in the implementation of EPHS

Political situation and stability	Afghanistan	Ethiopia	Pakistan	Somalia	Sudan	Zanzibar
	Fragile and conflict affected	Fragile and conflict affected	Politically volatile	Fragile and conflict affected	Post conflict, politically volatile	Political stable
**Types of private providers and policy framework towards PHS**						
Types of PHS providers and their contribution to delivery of services	Benefit packages provided by NGOs through contracting out to some not-for-profit hospitals Unqualified and underqualified providers mainly in rural areas Limited corporate commercial hospital sector	Formally registered providers, corporate commercial sector and some practitioners of traditional medicine	Private providers include qualified GPs, secondary and tertiary hospital, unqualified or underqualified, providers Some private hospitals empanelled and implement national health insurance packages	NGOs are the largest service providers Large number of unqualified providers, faith-based healers Small to medium secondary and tertiary hospitals exist mainly in urban areas	Many qualified small-to-medium private facilities Corporate sector has presence in cities Faith healers, herbal medicine sellers, traditional healers also provide services Many health facilities run by charities and not-for-profits	Most providers are registered, licensed, and monitored Some unqualified practitioners work as traditional healers. Private health facilities empanelled with national health insurance programme implement benefit packages
Policy framework towards the PHS	National policy on PHS exists There is a PHS oversight authority in the MOPH	Policy and legal framework for PHS exists and enforced by regulatory agency Licensing and registration system present for providers	PHS included in various policies Common regulatory framework for public and PHS facilities and providers implemented by licensing and registration bodies	No specific policy for the PHS, general law governs commercial sector National health sector strategy recognises PHS, no regulatory authority	No national policy framework or regulatory body for PHS Registration and licensing systems exist under different boards or bodies	Policy exists but no legal framework for PHS Private Hospital Advisory Board and professional bodies register facilities and providers
Current level of relationship/partnership between the public and PHS	PPP policy on health is managed by Ministry of Finance BPHS/EPHS are provided countrywide by NGOs via a contracting out model	Policy on engaging PHS for service delivery in cities PHS provides some services included in the EPHS but not obliged to do so Some pilot and small-scale PPPs and PHS engagement unit in exist in MoH	Some provinces and programmes have PPP policies for service develop and facility management through contracting to NGOs Contracts not always done through open competition or tied to results-based investments	Strategy exists for contracting service delivery to NGOs, private hospitals All NGOs required to deliver EPHS regardless of who contracts them MOH capacity to manage contracts is limited	No existing policy for PHS engagement, currently being developed by the federal MOH	MOH has a policy on PHS engagement for service delivery and some programmes and NGOs have PPP policies for health Private Hospital Advisory Board acts as the link between the MoH and the private sector
**Financial and service contribution of the PHS to the delivery of services**						
Private expenditure as % of THE	75% (2009) 77% (2019)	–	56.8%*	–	70.3%†	–
Out-of-pocket as % of THE	77%‡	–	51.9%*	47%	66.95%†	16%
Prepaid plans+social security as % of THE	Negligible	–	0.9%*	2%	SHI as % of THE=6.43%; SHI as % of GGHE 24.62%†	
PHS % annual total outpatient visits	NA	–	75%–80% mainly curative services	60% services provided by the PHS	NA	47% services by PHS mainly is curative
PHS % of inpatient episodes or hospital visits (year)	NA	–	NA; Bed density in PHS<3/10 000; public sector 6/10 000	–	NA	
**Policies and interventions used or piloted to engage the PHS in delivering health services and/or essential packages of health services**						
Outsourcing/contracting out	Delivery of BPHS and EPHS	None	Provinces contract out for delivery of primary and secondary services Provincial EPHS exist, role of contracting not defined	Private-for-profit sector provides more services not included in EPHS particularly in curative and rehabilitative care	NHIF contracts with private facilities to deliver listed services Newly defined EPHS co-developed by NHIF and FMoH and will be linked	NHIF, Jubilee and Strategies insurance company contracts with private facilities to deliver services
Social marketing or franchising	Limited vertical projects on family planning, iodised salt, ORS, iron and folic acid	Some	Mainly in family planning through donor funding	Not included in the health benefit package	Role of social marketing and franchising included in benefit package	NA
Social (health) insurance	NA	No	Sehat Sahulat programme (health insurance programme) covers selected inpatient tertiary and secondary services	Not included in the health benefit package	SHI covers >82% population with own list of services and medicines§	NA
Demand side interventions (vouchers or cash transfers)	Pilot projects for RMNCH services in two provinces	No	Limited and mainly from NGOs, mostly through direct donor financing	Not included in the health benefit package	With support from WB and EU, cash transfers done interruptedly in some areas	NA

*National Health Accounts 2017–2018.

†Sudan System of Health Accounts 2018.

‡National Health Accounts 2019.

§National Health Insurance Annual Report 2021.

BPHS, benefit package of health services; EPHS, essential package of health services; EU, European Union; FMoH, Federal Ministry of Health; GGHE, general government health expenditure; GPs, General Practitioners; MOH, Ministry of Health; MOPH, Ministry of Public Health; NA, not available; NGOs, non-governmental organisations; NHIF, National Health Insurance Fund; ORS, oral rehydration salts; PHS, private health sector; PPP, public–private partnership; RMNCH, reproductive, maternal, newborn and child health; SHI, social health insurance; THE, total health expenditure; WB, World Bank.

## Key challenges to engagement of the PHS: implications for EPHS implementation

There are multiple challenges to engaging the private sector in providing high-quality services as part of EPHS implementation. The first is incomplete information to understand the complexity and heterogeneity of private providers, which is a prerequisite for devising a clear role for these providers in implementing an EPHS. Second, private health providers are part of complex mixed health systems and need to complement the public sector without operating in isolation. The various roles that the private sector play in mixed health systems are elaborated in box 1.^12^

Box 1 **Categories of mixed health systems in low-income and lower middle-income countries and the role of the private sector**

1. In countries such as India and Nigeria, health systems are characterised by dominant private provision in primary and secondary care accompanied by high out-of-pocket (OOP) expenditure. Public expenditure on health is low, thus fees and other charges in the public sector create an additional access barrier prompting people to turn to private services, which include low quality unlicensed providers.
2. Countries such as Tanzania, Nepal, Ghana and Malawi show a stratified private health system with high OOP expenditure driven by private hospitals and clinics for the better off and extensive use of medicine selling in private shops by the poor. The public sector is characterised by varying levels of reliance on fees, which acts as a barrier to access, especially for the poor.
3. Countries such as Argentina and South Africa have a high-cost private health sector (PHS) used predominantly by affluent patients, which is largely financed by private health insurance. The poor generally rely on the public sector, where there is little reliance on service charges.
4. In Sri Lanka and Thailand, the private sector complements a universalist public sector. Well-funded, high-quality public health systems limit the private sector to a complementary role. This keeps OOP costs in check, which are mainly related to use of private services.
5. In transitioning systems, such as China, there is a small PHS. Traditionally, there is high private expenditure due to a commercialised public sector, but this is falling due to ongoing reforms.

Third, equity and population coverage become a challenge when the PHS is left to its own choices. Without any public subsidy, it generally provides only a limited set of services and crucial public health services are neglected. Private providers therefore are not geared to provide universal coverage of needed services even at the primary level without clear financing mechanisms, additional incentives and performance monitoring.^11^ Fourth, there are challenges related to quality and performance. It is often asserted that people use health services from the private sector because of better perceived quality compared with the public sector.^34^ However, perceived quality is often confused with technical quality and patient outcomes. In many cases, overall services are of low quality in both public and private sector.^35^ The final challenge relates to system inefficiency. Private health services may add to the overall costs of care through, for example, overuse of diagnostic services and expensive medications leading to waste of resources and other system inefficiencies such as antibiotic resistance. For routine and simple ailments, the public sector is more efficient by limiting overuse of resources and treatments and by providing preventive and public health services.^35^

## Leveraging the private sector to achieve UHC: what can governments do?

There is no denying the importance of engaging private health providers in the implementation of UHC packages in the context of LLMICs. What is less clear is how to do so, as the evidence is rather limited. Summarised below are the associated challenges and opportunities based on country experiences and possible options for governments to consider while implementing EPHS in partnership with the PHS.

Characterising private providers is essential to understanding their composition, characteristics and contribution to the overall provision of healthcare and in determining how the private sector will behave and respond to regulatory tools, incentives and disincentives, and market supply and demand dynamics. In systems where the public sector is inadequate and/or of low quality, engaging the private sector in delivering EPHS seems a realistic option—at least in the short to medium term—for rapidly improving access to essential health services and enhancing financial protection.^36 37^ Such engagement has its challenges related to governance issues, such as dual practice of health providers,^34^ poor quality of care, regulatory compliance and limited number of private service providers creating a barrier to the rapid increase in access to services.

One of the key take-aways is that while private providers have an important role to play in these contexts, they are not a panacea to the problem of limited, poor-quality access to healthcare services.^12 37^ For instance, the current evidence is mixed whether financial protection will be provided when services are offered by the private sector as part of a publicly funded benefit package.^34 36^ Although the private sector may play a significant role in the delivery of a publicly financed EPHS, concurrent improvement in the quality of public sector healthcare delivery in strategic and planned ways is an imperative. Whatever strategies are used to involve the private sector in the delivery of UHC packages, it is necessary to pay attention to the issues of performance and quality. Various regulatory tools such as credentialing, accreditation and use of key performance indicators in purchasing interventions from the private sector along with regular monitoring and enforcement will be needed.^37^

Several strategies can be used for regulating private providers such as better statutory control to prevent unlicensed practice, self-regulation by professional bodies to maintain professional standards of practice, and accreditation (especially of large private hospitals and chains). Additionally, purchasing delivery of essential services by engaging private providers can serve as an effective ‘regulatory tool’ to modify provider behaviour.

Large-scale purchasing of interventions has mainly been used in postconflict situations. While this may be a useful strategy to quickly increase access to services, its long-term sustainability is questionable, especially as donor interest fades over time.^33 36^ In Lebanon, the key challenges to contracting were a weak enabling environment, weak clinical governance and poor marketing and promotion of the package.^36^ In Egypt, PPPs have been used to deliver services for the basic package of health services for child and maternal care, primary care and laboratory services, directly managed by a Family Health Fund. In Pakistan, contracting with private providers has been used to improve access to services in remote areas or to improve the functionality of existing public sector facilities.^34^ However, the evidence for whether such efforts improve access and quality of services is mixed even for small portions of services.^17^ For contracting to be sustainable requires building capacity of the local governments to take over, having collaborative planning and review processes and involving key partners including community stakeholders in planning and monitoring.^38^ Further, assessment of cost-saving and value-for-money is key for financial sustainability, and should be part of monitoring and evaluation frameworks of such interventions.^39^ Given the unpredictability of global aid flows for health, governments in LLMICs need to increasingly rely on generating domestic revenues and using them efficiently.

Evidence for financial protection is also not clear. In Nigeria and Argentina, the adequacy of funds has been a problem, only a limited set of services could be provided, and financial sustainability of purchasing interventions has been questioned. In addition, most contracting initiatives in many LLMICs have not had a pro-poor focus, which suggests inadequate emphasis on equity.^34^ Therefore, given the evidence so far, it is not clear that large-scale purchasing could be an effective, efficient or sustainable strategy to provide the larger number of services included in an EPHS.

One view is that a package can be a tool or instrument of systematising and aligning the interests of private providers with the overall goals of the health system. In turn, the package can be leveraged as a coordination tool for organising the healthcare system and its components, such as financing, purchasing, provider payments and the organisation of service delivery, conceptualising the role of the private sector within this framework. The explicit nature of the package also facilitates negotiation and conditions of contracts between providers and the government.^40^

While incentives to providers are not always explicitly aligned with EPHS, in some countries there is evidence that purchasing strategies are used to ensure quality and efficiency in delivery of the packages. For instance, in Argentina, resources are linked to prioritised services and the outcomes obtained by the providers. Whereas in Mexico, where resources to providers are not linked with the services in EPHS, providers have limited incentives to provide services included in the package.^40^

Given the urgency to meet the UHC goals, what can governments do to navigate the challenges of implementing EPHS and progressively achieving UHC, and enhancing health security in the postpandemic scenario, while dealing with the uncertainty that is inherent in working with large, heterogenous, insufficiently documented and poorly regulated PHS? First, policy-makers need to characterise and understand the PHS in terms of service mix, health expenditure, distribution of services, as well as its interactions with the public sector as a prerequisite to its involvement in implementation of the EPHS. Second, attention must be paid to the supply side, especially the availability of health providers of various categories as that can limit their role in rapid expansion of service delivery. Third, a systematic preassessment of private providers and facilities should be conducted to identify any shortfalls in infrastructure and personnel needed to provide the services included in the EPHS.^16^ Delivery of EPHS will not be realised unless these gaps in health systems are systematically identified and addressed.^3^ Fourth, investment and capacity building will also be needed in developing high-quality contract management monitoring and enforcement systems. Finally, increase in overall health expenditure is a must for effective engagement of the private sector in EPHS implementation.

The health systems in three of the six countries assessed have been devastated by conflict, political instability and underinvestment. They face unique challenges of coordinating and dealing with the fragmented aid system accompanied by large number of NGOs supported by donors who come with different approaches to planning, financing, implementing, monitoring and evaluation. The governance system in those countries has either collapsed or been severely weakened and financing healthcare largely depends on foreign aid. Yet, opportunities also exist to rebuild their health systems, including the options on models of service delivery, for example, the adoption of public financing and private provision. Recent experience in these countries shows that policy-makers are more receptive to positive change than one would expect to encounter while transforming rigid and unyielding health systems.

## Conclusion

In systems where the PHS currently provides a substantial proportion of services and where public sector is inadequate and/or of low quality, providing EPHS without involvement of the PHS is unrealistic at least in the short term. It is in these systems that institution and reliable delivery of EPHS through the involvement of the PHS is most likely to be of benefit in rapidly improving access to essential services and financial protection.

While PHS involvement in UHC is inevitable, the challenges that surround its engagement need to be taken into cognisance before a coherent strategy is formulated by the countries towards PHS engagement. More research will be needed to better explore the role of the PHS in implementing EPHS. Some of the recommended options can be operationalised by developing a guide for engaging the PHS, which can be adapted to the local context; and by piloting EPHS implementation at small subnational administrative levels, for example, by conducting a cluster randomised trial in a district and assessing impact and providing recommendations for scaling up implementation of EPHS for UHC.

## Data Availability

No data are available.

## References

[1] World Health Organization. Universal health coverage. Available: https://www.who.int/health-topics/universal-health-coverage#tab=tab_1 [Accessed 16 Nov 2022].

[2] Wright J, Holtz J. Essential packages of health services in 24 countries: findings from a Cross-Country analysis, 2017. USAID. Available: https://www.hfgproject.org/ephs-cross-country-analysis/ [Accessed 16 Nov 2022].

[3] World Health Organization. Principles of health benefit packages: World Health organization, 2021. Available: https://www.who.int/publications/i/item/9789240020689 [Accessed 05 Nov 2022].

[4] Glassman A, Giedion U, Sakuma Y, et al. Defining a health benefits package: what are the necessary processes? Health Syst Reform 2016;2:39–50. 10.1080/23288604.2016.1124171 31514661

[5] World Health Organization. Making fair choices on the path to universal health coverage: final report of the who consultative group on equity and universal health coverage, 2014. World Health organization. Available: https://apps.who.int/iris/handle/10665/112671 [Accessed 12 Mar 2022].

[6] Gopinathan U, Ottersen T. Evidence-informed deliberative processes for Universal Health Coverage: broadening the scope comment on "priority setting for Universal Health Coverage: we need evidence-informed deliberative processes, not just more evidence on cost-effectiveness". Int J Health Policy Manag 2017;6:473–5. 10.15171/ijhpm.2016.148 28812847 PMC5553216

[7] Glassman A, Chalkidou K. Priority-setting in health: building institutions for smarter public spending. Washington, DC: Center for Global Development, 2012.

[8] Hallo De Wolf A, Toebes B. Assessing private sector involvement in health care and universal health coverage in light of the right to health. Health Hum Rights 2016;18:79. 28559678 PMC5394993

[9] Jansen MP, Baltussen R, Mikkelsen E, et al. Evidence-informed deliberative processes–early dialogue, broad focus and relevance: a response to recent commentaries. Int J Health Policy Manag 2018;7:96–7. 10.15171/ijhpm.2017.88 29325411 PMC5745876

[10] Horton R, Clark S. The perils and possibilities of the private health sector. Lancet 2016;388:540–1. 10.1016/S0140-6736(16)30774-7 27358254

[11] McPake B, Hanson K. Managing the public-private mix to achieve universal health coverage. Lancet 2016;388:622–30. 10.1016/S0140-6736(16)00344-5 27358252

[12] Mackintosh M, Channon A, Karan A, et al. What is the private sector? Understanding private provision in the health systems of low-income and middle-income countries. Lancet 2016;388:596–605. 10.1016/S0140-6736(16)00342-1 27358253

[13] Marten R, McIntyre D, Travassos C, et al. An assessment of progress towards universal health coverage in Brazil, Russia, India, China, and South Africa (BRICS). Lancet 2014;384:2164–71. 10.1016/S0140-6736(14)60075-1 24793339 PMC7134989

[14] Stallworthy G, Boahene K, Ohiri K, et al. Roundtable discussion: what is the future role of the private sector in health? Global Health 2014;10:1–5. 10.1186/1744-8603-10-55 24961806 PMC4094675

[15] Tung E, Bennett S. Private sector, for-profit health providers in low and middle income countries: can they reach the poor at scale? Global Health 2014;10:52–9. 10.1186/1744-8603-10-52 24961496 PMC4094686

[16] Partnership for Maternal, Newborn & Child Health (PMNCH), Swedish International Development Cooperation Agency (SIDA). Prioritizing essential packages of health services in six countries in sub-Saharan Africa: implications and lessons for SRHR, 2019. Available: https://evidentdesign.dk/wp-content/uploads/2020/06/53306-WHO-One-PMNCH-report-web.pdf [Accessed 25 Aug 2022].

[17] Odendaal WA, Ward K, Uneke J, et al. Contracting out to improve the use of clinical health services and health outcomes in low- and middle-income countries. Cochrane Database Syst Rev 2018;4:Cd008133. 10.1002/14651858.CD008133.pub2 29611869 PMC6494528

[18] Palmer N, Strong L, Wali A, et al. Contracting out health services in fragile states. BMJ 2006;332:718–21. 10.1136/bmj.332.7543.718 16565130 PMC1410853

[19] Siddiqi S, Masud TI, Sabri B. Contracting but not without caution: experience with outsourcing of health services in countries of the eastern Mediterranean region. Bull World Health Organ 2006;84:867–75. 10.2471/BLT.06.033027 17143460 PMC2627537

[20] Newbrander W, Ickx P, Feroz F, et al. Afghanistan's basic package of health services: its development and effects on rebuilding the health system. Glob Public Health 2014;9 Suppl 1:S6–28. 10.1080/17441692.2014.916735 24865404 PMC4136668

[21] Bloom E, Bhushan I, Clingingsmith D. Contracting for health: evidence from Cambodia. unpublished working paper. Washington, DC: Brookings Institution, 2006.

[22] Vaux T, Visman E. Service delivery in countries emerging from conflict. London: Centre for International Co-operation and Security (CICS), Department of Peace Studies, University of Bradford, 2005.

[23] Islamic Republic of Afghanistan Ministry of Public Health. A basic package of health services for Afghanistan. Kabul Ministry of Public Health; 2009. https://extranet.who.int/mindbank/item/5613 [Accessed 05 Mar 2022].

[24] Loevinsohn B, Sayed GD. Lessons from the health sector in Afghanistan: how progress can be made in challenging circumstances. JAMA 2008;300:724–6. 10.1001/jama.300.6.724 18698072

[25] Asian Development Bank. Project completion report on the basic health services project in Cambodia, 2004. Available: https://www.adb.org/sites/default/files/project-document/70061/pcr-cam-27410.pdf [Accessed 29 Aug 2022].

[26] DCP3 country translation project. Available: http://dcp-3.org/translation [Accessed 29 Aug 2022].

[27] Alwan A, Majdzadeh R, Yamey G. Country readiness and prerequisites for successful design and implementation of essential packages of health services: experience from six countries. BMJ Glob Health 2022:[Under submission]. 10.1136/bmjgh-2022-010720 PMC9853149 36657808

[28] Baltussen R, Mwalim O, Blanchet K, et al. Decision-making processes for essential packages of health services: experience from six countries. BMJ Glob Health 2022:[Under submission]. 10.1136/bmjgh-2022-010704 PMC9853142 36657809

[29] Gaudin S, Raza W, Blanchet K, et al. Using costing to facilitate policy making toward UHC: findings and recommendations from country-level experiences. BMJ Glob Health 2022:[Under submission]. 10.1136/bmjgh-2022-010735 PMC9853124 36657806

[30] Soucat A, Tandon A, González-Pier E. From UHC health services packages to budget appropriation: the long journey to implementation. BMJ Glob Health 2022:[Under submission]. 10.1136/bmjgh-2022-010755 PMC10186405 37188361

[31] Reynolds T, Wilkinson T, Bertram MY. Building implementable packages for universal health coverage. BMJ Glob Health 2022:[Under submission]. 10.1136/bmjgh-2022-010807 PMC10201243 37197791

[32] Danforth K, Ahmad A, Blanchet K, et al. Monitoring and evaluating the implementation of essential packages of health services. BMJ Glob Health 2022:[Under submission]. 10.1136/bmjgh-2022-010726 PMC10069525 36977532

[33] Sabri B, Siddiqi S, Ahmed AM, et al. Towards sustainable delivery of health services in Afghanistan: options for the future. Bull World Health Organ 2007;85:712–8. 10.2471/BLT.06.036939 18026628 PMC2636401

[34] World Health Organization Regional Office for the Eastern Mediterranean. Role and contribution of private sector in moving towards universal health coverage in the eastern Mediterranean region, 2016. World Health Organization. Regional Office for the Eastern Mediterranean. Available: https://apps.who.int/iris/handle/10665/249567 [Accessed 29 Aug 2022].

[35] Morgan R, Ensor T, Waters H. Performance of private sector health care: implications for universal health coverage. Lancet 2016;388:606–12. 10.1016/S0140-6736(16)00343-3 27358251

[36] Ensor T, Dave-Sen P, Ali L, et al. Do essential service packages benefit the poor? Preliminary evidence from Bangladesh. Health Policy Plan 2002;17:247–56. 10.1093/heapol/17.3.247 12135990

[37] Montagu D, Goodman C. Prohibit, constrain, encourage, or purchase: how should we engage with the private health-care sector? Lancet 2016;388:613–21. 10.1016/S0140-6736(16)30242-2 27358250

[38] Strasser S, Stauber C, Shrivastava R, et al. Collective insights of public-private partnership impacts and sustainability: a qualitative analysis. PLoS One 2021;16:e0254495. 10.1371/journal.pone.0254495 34283847 PMC8291689

[39] Pinz A, Roudyani N, Thaler J. Public–private partnerships as instruments to achieve sustainability-related objectives: the state of the art and a research agenda. Public Manag Rev 2018;20:1–22. 10.1080/14719037.2017.1293143

[40] Giedion U, Bitrán R, Tristao I. Health benefit plans in Latin America: a regional comparison. Inter-American development bank. Spanish Planes de beneficios en salud de América Latina; 2014. https://www.idsihealth.org/wp-content/uploads/2015/01/Health-Benefit-Plans-in-Latin-America_IDB.pdf [Accessed 29 Aug 2022].

[41] Sundari Ravindran TK, Fonn S. Are social franchises contributing to universal access to reproductive health services in low-income countries? Reprod Health Matters 2011;19:85–101. 10.1016/S0968-8080(11)38581-3 22118144

[42] Tangcharoensathien V, Limwattananon S, Patcharanarumol W, et al. Achieving universal health coverage goals in Thailand: the vital role of strategic purchasing. Health Policy Plan 2015;30:1152–61. 10.1093/heapol/czu120 25378527 PMC4597041

